# Complete Genome Sequence of *Pseudomonas aeruginosa* Strain YL84, a Quorum-Sensing Strain Isolated from Compost

**DOI:** 10.1128/genomeA.00246-14

**Published:** 2014-04-03

**Authors:** Kok-Gan Chan, Wai-Fong Yin, Yan Lue Lim

**Affiliations:** Division of Genetics and Molecular Biology, Institute of Biological Sciences, Faculty of Science, University of Malaya, Kuala Lumpur, Malaysia

## Abstract

Here, we report the complete genome sequence of *Pseudomonas aeruginosa* strain YL84, which was isolated from compost. This strain was found to be a chitinase-producing quorum-sensing bacterium.

## GENOME ANNOUNCEMENT

*Pseudomonas aeruginosa*, which is from the *Pseudomonadaceae* family, is an aerobic, motile, and Gram-negative rod-shaped bacterium that exists in a wide range of ecological niches. *P. aeruginosa* is known to be a prevalent opportunistic human pathogen and is also one of the most important causative agents of hospital-acquired nosocomial infections, characteristically in immunocompromised individuals (1–3). The secretion of a vast multitude of virulence factors that are responsible for the diverse and overwhelming pathogenicity of *P. aeruginosa* is found to be controlled by a cell-to-cell signaling mechanism known as quorum sensing (1, 4).

Quorum sensing is a cell-density-dependent communication system that relies on *N-*acylhomoserine lactone as the signaling molecule. It is used by a large number of Gram-negative bacteria to regulate population behavior (5, 6). Two types of quorum-sensing systems have been described in *P. aeruginosa*, the *las* and *rhl* systems (7). Here, we report the complete genome sequence of a quorum-sensing *P. aeruginosa* strain, YL84, which was isolated from compost. Chitinase activity is also found in this isolate.

Genomic DNA of *P. aeruginosa* YL84 was extracted using a MasterPure DNA purification kit (Epicentre, Inc., Madison, WI). Appropriately sheared genomic DNA was used to construct a SMRTbell library using P4 chemistry. Whole-genome sequencing was subsequently performed using a Pacific Biosciences RSII sequencing platform (Pacific Biosciences, Menlo Park, CA). Four single-molecule real-time (SMRT) cells were used in the sequencing process, yielding an average genome coverage of 210.43×. Primary filtering of the sequenced data was done in the PacBio Blade Center, and the filtered data were subsequently processed in the SMRT Portal.

A total of 279,487 reads, with a mean read length of 5,261 bp, were obtained following the primary filtering. *De novo* assembly was performed using the hierarchical genome assembly process (11) (PacBio DevNet; Pacific Biosciences), which successfully assembled the genome into a single contig with a maximum contig length of 6,433,441 bp and an overall G+C content of 66.43%. The Rapid Annotations using Subsystems Technology (RAST) pipeline (8) was used to predict and annotate open reading frames (ORFs) of the genome, and 5,992 protein-coding ORFs with known protein functions were found to be present in the chromosome. ARAGORN (9) and RNAmmer (10) were used to identify tRNA and rRNA genes, respectively. From these analyses, 74 tRNAs and 12 rRNA operons, comprising four 5S, four 16S, and four 23S rRNA genes, were detected in the genome.

The presence of both chitinase and chitin-binding proteins was identified from the annotated genome. The chitinase is predicted to be a 480-amino-acid protein that consists of a GH18 catalytic domain, a fibronectin type III (Fn 3) domain, and a chitin-binding domain, whereas the chitin-binding protein (CBP), CbpD, is a 389-amino-acid protein that belongs to family 33 of CBPs.

### Nucleotide sequence accession number.

The results of this whole-genome shotgun project have been deposited at DDBJ/EMBL/GenBank under the accession no. CP007147.
